# Genome Sequences of Two *Brucella suis* Strains Isolated from the Same Patient, 8 Years Apart

**DOI:** 10.1128/genomeA.01687-16

**Published:** 2017-03-02

**Authors:** Marcus Vinicius Canário Viana, Alice Rebecca Wattam, Dhwani Govil Batra, Sébastien Boisvert, Thomas Scott Brettin, Michael Frace, Fangfang Xia, Vasco Azevedo, Rebekah Tiller, Alex R. Hoffmaster

**Affiliations:** a Laboratório de Genética Celular e Molecular, Universidade Federal de Minas Gerais, Belo Horizonte, Minas Gerais, Brazil; b Biocomplexity Institute of Virginia Tech, Virginia Tech University, Blacksburg, Virginia, USA; c Centers for Disease Control and Prevention, Atlanta, Georgia, USA; d Gydle, Inc., Québec, Québec, Canada; e Computing, Environment and Life Sciences, Argonne National Laboratory, Argonne, Illinois, USA; f Computation Institute, University of Chicago, Chicago, Illinois, USA; g Biotechnology Core Facility Branch, National Center for Emerging and Zoonotic Infectious Diseases, Centers for Disease Control and Prevention, Atlanta, Georgia, USA; h Mathematics and Computer Science Division, Argonne National Laboratory, Argonne, Illinois, USA; i Bacterial Special Pathogens Branch, Division of High-Consequence Pathogens and Pathology, Centers for Disease Control and Prevention, Atlanta, Georgia, USA

## Abstract

*Brucella suis* is a Gram-negative, facultative intracellular pathogen that has pigs as its preferred host, but it can also infect humans. Here, we report the draft genome sequences of two *B. suis* strains that were isolated from the same patient, 8 years apart.

## GENOME ANNOUNCEMENT

Brucellosis is the most common zoonosis worldwide caused by bacteria of the *Brucella* genus, transmitted by direct or indirect contact with infected animals or their products. Its chronic infection causes infertility and abortion in animals, which is not only a burden for the animals but also has an economic impact. In humans, infection with *Brucella* causes an acute or subacute febrile illness, along with varied and nonspecific clinical manifestations that include fever, sweats, fatigue, malaise, anorexia, weight loss, headache, arthralgia, and back pain (1). *Brucella* is part of the family *Brucellaceae*, order *Rhizobiales*, and class *Alphaproteobacteria* (2). All members of this genus are Gram-negative, facultative intracellular pathogens, and 10 species have been described that are differentiated by the mammalian host that they prefer to infect, as well as a set of antigenic and metabolic phenotypes (3). *Brucella* spp. cause long-term chronic infections as intracellular pathogens, and they are noted as having reduced genomes, a type IV secretion system, a perosamine-based O antigen, and reduced virulence (4).

*Brucella suis* generally is found to infect pigs, but it can also infect other animals, including humans. Although *B. melitensis* is the most common species causing human disease*, B. suis* also commonly affects them and is the most common *Brucella* sp. to infect people in Argentina (5, 6).

*B. suis* strains 2004000577 and 2011017258 were isolated from a recrudescent case from a single human patient but were collected 8 years apart (2003 and 2011, respectively) in Massachusetts, USA. Both genomes were collected from the same leg wound, the first collection in October 2003, and the second in April 2011. The genomes were sequenced using the Roche 454 platform. For strain 2004000577, a *de novo* assembly was performed with Newbler version 2.9. Scaffolding was done with CONTIGuator version 2.7, using *B. suis* 1330 (AE014291.4, AE014292.2) as the reference genome. Gaps closure was performed by reference assembly (7), using CLC Genomics Workbench version 6.5 (Qiagen, USA) and *B. suis* 1330 as the reference genome. For strain 2011017258, a reference assembly was done by mapping the sequencing reads to the *B. suis* 1330 (AE014291.4, AE014292.2) genome sequence. Automatic annotation was done by RAST*tk* (8) at the PATRIC bioinformatics resource center (9, 10).

Strain 2004000577 had a total of 137,159 reads and a mean coverage depth of 15.98×. The complete genome of strain 2004000577 has two chromosomes with a total size of 3,315,231 bp, 3,329 coding sequences (CDSs), 55 tRNA genes, nine rRNA genes, and a G+C content of 57.25%.

Strain 2011017258 had a total of 303,995 reads and a mean coverage depth of 11.52×. The reference assembly resulted in scaffolds of chromosome I and II, with 36 and 35 contigs, respectively, with a total of 3,317,988 bp. The draft genome of strain 2011017258 has 4,063 CDSs, 55 tRNA genes, nine rRNA genes, and a G+C content of 57.21%.

### Accession number(s).

The genome sequences reported here have been deposited in GenBank under the accession numbers CP016981 and CP016982 for *B. suis* 2004000577 and CP017012 and CP017013 for *B. suis* 2011017258.
